# Real-world patient characteristics and treatment outcomes among nontransplanted multiple myeloma patients who received Bortezomib in combination with Lenalidomide and Dexamethasone as first line of therapy in the United States

**DOI:** 10.1186/s12885-022-09980-9

**Published:** 2022-08-18

**Authors:** Rohan Medhekar, Tao Ran, Alex Z. Fu, Sharmila Patel, Shuchita Kaila

**Affiliations:** 1 Janssen Scientific Affairs, LLC, 800 Ridgeview Drive, Horsham, PA 19044 USA; 2 Georgetown University Medical Center, 37th and O Street, NW, Washington, DC 20057 USA

**Keywords:** Newly diagnosed multiple myeloma, Bortezomib, Lenalidomide, Dexamethasone, Real-world evidence

## Abstract

**Background:**

There is limited real-world evidence that describes patients with newly diagnosed multiple myeloma (NDMM) treated with the bortezomib, lenalidomide, and dexamethasone (VRd) triplet regimen. We evaluated patient characteristics and treatment outcomes among nontransplanted NDMM patients who received VRd as their first line of therapy (LOT) in US oncology practice settings.

**Methods:**

This retrospective observational cohort study evaluated patients from the Flatiron MM Core Registry who received VRd as first LOT between November 1, 2015, and February 28, 2021. Progression-free survival (PFS) was analyzed using the Kaplan-Meier method. Associations between patient demographic and clinical characteristics and PFS were evaluated using a multivariable Cox proportional hazards model.

**Results:**

A total of 2342 eligible patients with VRd as first LOT were identified (mean age, 67.0 years). Among all identified patients, 64.3% were ≥ 65 years of age, 25.5% were elderly (≥75 years), and 47.9% were frail. Among patients with available data, 21.2% had high-risk cytogenetics, and the majority had International Staging System (ISS) stage I/II disease (71.8%), and Eastern Cooperative Oncology Group performance status (ECOG PS) score 0/1 (81.2%). Median duration of therapy was 5.5 months. With median follow-up of 21.0 months, median PFS and time-to-next-treatment were 26.5 and 16.1 months, respectively. Higher risk of disease progression or death was seen in patients categorized as elderly (hazard ratio [HR] = 1.37; 95% confidence interval [CI]: 1.13-1.66 vs patients < 65 years), having high-risk cytogenetics (HR = 1.44; 95% CI: 1.19-1.75 vs standard risk), having ISS disease stages II and III (HR = 1.31; 95% CI: 1.06-1.63 and HR = 1.37; 95% CI: 1.10-1.70 versus stage I, respectively), and having worse ECOG PS score (≥2) (HR = 1.49; 95% CI: 1.22-1.81 versus functionally active patients).

**Conclusions:**

The majority of patients treated with VRd in this study were ≥ 65 years of age, were ISS stage I/II, had an ECOG PS score of 0/1, and had standard cytogenetic risk. Median PFS observed in real-world practice was notably shorter than that observed in the SWOG S0777 clinical trial. In nontransplanted patients treated with VRd as first LOT, a higher risk of disease progression or death was associated with older age, having high-risk cytogenetics, worse disease stage, and worse ECOG PS score.

**Supplementary Information:**

The online version contains supplementary material available at 10.1186/s12885-022-09980-9.

## Background

Multiple myeloma (MM) is a hematological malignancy that is characterized by clonal proliferation of malignant plasma cells within the bone marrow [1]. Data from the US Surveillance, Epidemiology, and End Results registry estimate an annual incidence of 7.1 per 100,000 persons per year and a 5-year survival rate of 56% [2]. High-dose chemotherapy followed by autologous stem cell transplantation is considered the standard of care in younger and fit older patients with newly diagnosed multiple myeloma (NDMM) [3, 4]. Older patients with substantial comorbidities may be unable to tolerate such treatment; accordingly, although consensus and practice may vary somewhat by region, these patients may be ineligible for stem cell transplantation if they are deemed to be elderly or frail with comorbidities [3–5].

In the United States, the most common regimens used as first line of therapy (LOT) for transplant-ineligible patients with NDMM include lenalidomide in combination with dexamethasone (Rd), bortezomib in combination with lenalidomide and dexamethasone (VRd), and bortezomib in combination with dexamethasone (Vd) [6]. In recent years, the NCCN Clinical Practice Guidelines in Oncology (NCCN Guidelines®) [7] and the general treatment paradigm have been shifting toward triplet regimens. The VRd regimen has become one of the preferred regimens in transplant-ineligible patients with NDMM, and sufficient fitness for triplet therapy based on the results from the phase 3 Southwest Oncology Group (SWOG) S0777 study was associated with superior progression-free survival (PFS), overall survival, and overall response rate with VRd over Rd in NDMM patients without intent for immediate transplant [8, 9]. Notably, the treatment landscape for this patient population continues to evolve; for instance, based on the results of the recent phase 3 MAIA study [10], daratumumab in combination with lenalidomide and dexamethasone (D-Rd) has also been recommended by the NCCN guidelines as a preferred regimen in transplant-ineligible patients with NDMM [7].

Although the efficacy of VRd in NDMM was demonstrated in SWOG S0777 [8, 9], clinical trials have strict eligibility criteria that may not translate to the real-world setting. Of note, in the VRd arm of the SWOG S0777 study only 28 (11%) patients were > 75 years of age and 56 (21%) patients were considered frail [11]. Patients with comorbidities such as renal impairment and cardiovascular disease are commonly excluded from clinical trials, likely contributing to some observations of better survival outcomes among patients who participate in those trials than in patients treated in the real-world setting [12, 13]. Given limited real-world data on the characteristics and outcomes of patients with NDMM treated with VRd, especially older, frail patients with comorbidities, we conducted a study to address these knowledge gaps and to supplement clinical trial data.

The aim of this study was to evaluate patient characteristics and treatment outcomes (PFS and time-to-next–treatment) among NDMM patients who received VRd as first LOT in US oncology practice settings. Associations between baseline patient characteristics and PFS were also evaluated.

## Methods

### Study design and data sources

This retrospective, observational, cohort study utilized the patient data obtained from the Flatiron MM Core Registry to select NDMM patients treated with VRd as first LOT from November 1, 2015, to February 28, 2021. The Flatiron Health electronic health record (EHR)–derived database provides deidentified patient-level data for patients treated at community oncology practices and academic medical centers across the United States. Flatiron processes both structured data (data points that are organized in a predefined manner, such as drop-down fields that reside in an EHR to capture a patient’s gender or date of birth) and unstructured data (information that is not organized in a preexisting data model, such as free text from a physician note or lab report). The entire patient chart from the EHR is available for each patient treated in the Flatiron network. The Flatiron database contains approximately 2.2 million active patient records, with approximately more than 13,000 patients with recorded diagnoses of MM as of the data cutoff date.

The Flatiron MM Core Registry included patients with the following: a diagnosis of MM (International Classification of Disease, 9th Revision, Clinical Modification [ICD-9-CM] diagnosis code 203.0x or International Classification of Disease, 10th Revision, Clinical Modification [ICD-10-CM] diagnosis code C90.0x coded in structured EHR data) between January 1, 2011, and February 28, 2021; ≥2 visits at a clinic contributing data to Flatiron between January 1, 2011, and February 28, 2021 (a probabilistic sample of MM patients meeting the first 2 criteria was selected by Flatiron for abstraction of unstructured chart data including physician notes, pathology reports, radiology reports, and discharge summaries); physician diagnosis of active MM confirmed via chart abstraction; and initial MM diagnosis date ≤90 days prior to start of structured data in Flatiron EHR. The first observed date of MM diagnosis was designated as the diagnosis date. The index date was defined as the first observed record of the VRd regimen. A LOT, defined as the first administration or non-cancelation of drugs given ≤28 days apart, was started on or after the first day of an administration or non-cancelation of an MM regimen given after or up to 14 days before the diagnosis date and after the start of structured activity. A gap of ≤90 days was allowed within a LOT.

The confidentiality of all patient records was maintained during the study. All information on individual patients was deidentified, and no patient-identifying information was provided to investigators.

### Patients

Patients with MM, without initiation of therapy prior to the diagnosis date, and with VRd as first LOT between November 1, 2015, and February 28, 2021, were selected from the Flatiron MM Core Registry. Patients were included if their first LOT initiation date was ≤30 days prior to start of structured data in Flatiron EHR. Nontransplanted patients were defined as those who did not receive a hematopoietic stem cell transplant (HSCT) from the diagnosis date to the index date. Patients were excluded from the analysis if they were < 18 years of age on the index date; were enrolled in a clinical trial on the index date; had other malignancies prior to the index date; or had a diagnosis of amyloid light-chain amyloidosis (ICD-9 CM, 277.39; ICD-10 CM, E85.81) prior to the index date.

### Assessments

Demographics, clinical characteristics, and treatment and outcome measurements were assessed in the full study cohort. Clinical characteristics assessed included the proportions of patients categorized as elderly (≥75 years of age on the index date); frail (with frailty score ≥ 2 calculated using age on the index date, Charlson Comorbidity Index during the entire pre-index period, and Eastern Cooperative Oncology Group performance status [ECOG PS] score on the closest date to the index date ≤90 days prior to and ≤ 7 days after the index date; Supplementary Table 1) [14]; having acute renal impairment (patients with a serum creatinine > 2 mg/dL on the closest date to the index date or having diagnosis codes of ICD-9-CM 584.5-584.9, ICD-9-CM 586, ICD-10-CM N17.0-2, N17.8-9, and N19, measured ≤90 days prior to and ≤ 7 days after the index date); and having diabetes (patients with diagnosis codes of ICD-9-CM 250.x or ICD-10-CM E10.x-E11.x prior to the index date). International Staging System (ISS) disease stage was measured on the initial diagnosis date. ECOG PS score was measured on the closest date to the index date ≤90 days prior to and ≤ 7 days after the index date. Cytogenetic risk (with high-risk cytogenetics defined as the presence of t[4;14], t[14;16], and/or del17p abnormalities) was measured on the test date via fluorescence in situ hybridization. Creatinine clearance, calculated using the Cockcroft-Gault formula, and CRAB (hypercalcemia, renal impairment [RI], anemia, and bone disease) symptoms were measured on the closest date to the index date ≤90 days prior to and ≤ 7 days after the index date. Duration of therapy was defined as the time between the index date and the last episode date of the last drug within the LOT. Follow-up time was defined as the time between the index date and the patient’s last activity date. Time-to-next-treatment was defined as the time interval between the index date and the start of the next LOT or death, whichever occurred first. PFS was measured as the time interval between the index date and disease progression or death, whichever occurred first. Disease progression was defined as a clinically meaningful increase in serum M-protein, urine M-protein, or the free light chain ratio according to the International Myeloma Working Group criteria [15–17].

### Statistical analyses

Continuous variables were presented descriptively as the mean, standard deviation (SD), or median, and categorical variables were presented as frequency and percentage. Time-to-event variables were summarized using the Kaplan-Meier method. Patients were censored on the end-of-therapy date for the duration of therapy unless they had a clinic visit ≥120 days post index date, or experienced death or next LOT or HSCT. Patients were also censored if they reached the date of their last confirmed activity, or if they reached the end of the data cut (whichever occurred first) for time-to-next–treatment; or if they reached the relevant last lab date or the end of the data cut (whichever occurred first) for PFS. In addition, patients who had post-index HSCTs before the time-to-next-treatment or PFS events were also censored. Percentage of missingness was reported for each variable. Median time-to-next-treatment and PFS were reported. A multivariable Cox proportional hazards model was used to evaluate the association between the following demographic and clinical characteristics and PFS: age (< 65, 65 to < 70, 70 to < 75, ≥75), race (White/Black [or African American]/Asian or other race), gender, cytogenetic risk (high/standard), diabetes (yes/no), RI (yes/no), ISS disease stage (I, II, III), ECOG PS score (0, 1, ≥2), year of index date, and time from initial diagnosis to index date. Because frailty was defined using age and ECOG PS score, it was expected to be highly correlated with these variables and hence was excluded from multivariable analysis. Results are presented as adjusted hazard ratios (HRs) with associated 95% confidence intervals (CIs). Prior to running the Cox proportional hazards model, multiple imputation was used as the primary approach to handle missing values.

## Results

### Patient characteristics

**Fig. 1:**
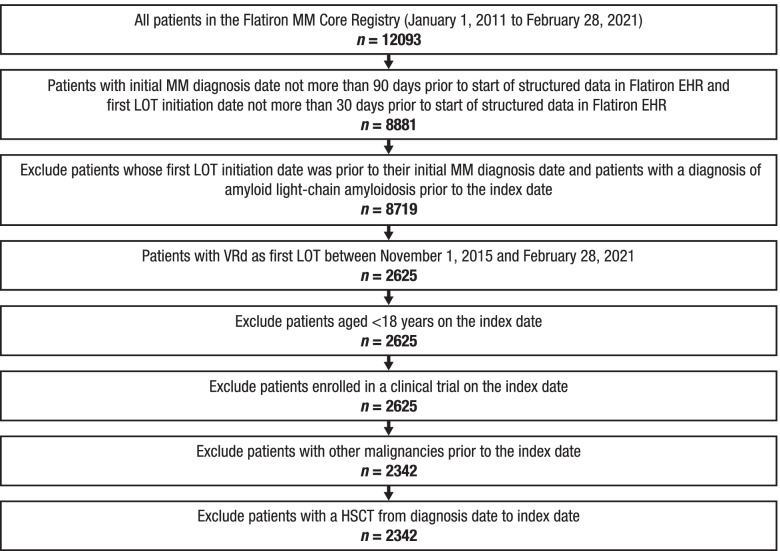
Attrition of patients by eligibility criteria MM multiple myeloma, EHR electronic health record, LOT line of therapy, VRd bortezomib, lenalidomide, and dexamethasone, HSCT hematopoietic stem cell transplant

A total of 2342 patients were identified who received VRd therapy as first LOT between November 1, 2015, and February 28, 2021 (Fig. 1). Among all identified patients, the mean age at the index date was 67.0 years (SD, 10.0), 64.3% were ≥ 65 years of age, 25.5% were elderly (≥75 years of age), slightly over half (53.3%) were male, 58.2% were White, 47.9% were frail, and 10.7% had RI (Table 1**)**. Among patients with available data, the majority had an ISS disease stage of I or II (71.8% [1133/1577]), an ECOG PS score of 0 or 1 (81.2% [1488/1832]), and standard-risk cytogenetics (78.8% [1393/1767]).

**Table 1:** Patient demographic and clinical characteristics among all patients identified

Characteristic	VRd as first LOT (***N*** = 2342)
Age at index date, years, mean (SD)	67.0 (10.0)
Age group, years, n (%)^a^	
< 65	836 (35.7)
65 to < 70	466 (19.9)
70 to < 75	443 (18.9)
≥ 75	597 (25.5)
Sex, n (%)^a^	
Female	1094 (46.7)
Male	1248 (53.3)
Race, n (%)^a^	
White	1363 (58.2)
Black or African American	411 (17.5)
Asian	51 (2.2)
Other	282 (12.0)
Unknown	235 (10.0)
ISS disease stage, n (%)^a^	
I	580 (24.8)
II	553 (23.6)
III	444 (19.0)
Unknown	765 (32.7)
ECOG PS score, n (%)^a^	
0	770 (32.9)
1	718 (30.7)
2	261 (11.1)
3	80 (3.4)
4	3 (0.0)
Unknown	510 (21.8)
Cytogenetic risk, n (%) ^a^	
High	374 (16.0)
Standard	1393 (59.5)
Unknown	575 (24.6)
Frail, n (%)^a^	1122 (47.9)
Patients with CRAB symptoms, n (%)^a^	
Any	1175 (50.2)
Hypercalcemia	232 (9.9)
RI^b^	250 (10.7)
Anemia	989 (42.2)
Bone disease	31 (1.3)
All	2 (0.1)
Year of index date, n (%)^a^	
2015	31 (1.3)
2016	410 (17.5)
2017	452 (19.3)
2018	495 (21.1)
2019	520 (22.2)
2020	388 (16.6)
January to February 2021	46 (2.0)
Diabetes, n (%)^a^	211 (9.0)

*VRd* bortezomib, lenalidomide, and dexamethasone, *LOT* line of therapy, *SD* standard deviation, *ISS* International Staging System, *ECOG PS* Eastern Cooperative Oncology Group performance status, *CRAB* hypercalcemia, renal impairment, anemia, and bone disease, *RI* renal impairment, *ICD-9-CM* International Classification of Disease, 9th Revision, Clinical Modification; *ICD-10-CM* International Classification of Disease, 10th Revision, Clinical Modification

^a^Percentages are calculated among all 2342 patients identified as receiving VRd as first LOT. Percentages may not add to 100 due to rounding

^b^Acute RI, defined as patients with a serum creatinine > 2 mg/dL on the closest date to the index date or having diagnosis codes of ICD-9-CM 584.5-584.9, ICD-9-CM 586, ICD-10-CM N17.0-2, N17.8-9, and N19, measured ≤90 days prior to and ≤ 7 days after the index date

### Treatment outcomes

**Fig. 2:**
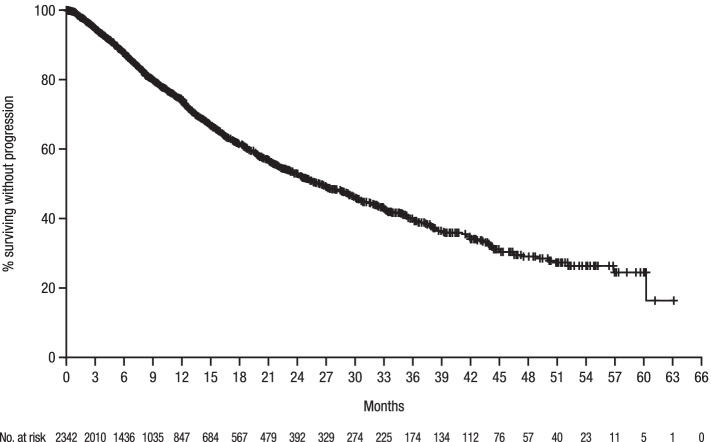
PFS for nontransplanted NDMM patients treated with VRd as first LOT PFS progression-free survival, NDMM newly diagnosed multiple myeloma, VRd bortezomib with lenalidomide and dexamethasone, LOT line of therapy

The median follow-up was 21.0 months. The median duration of therapy was 5.5 months. Median time-to-next–treatment was 16.1 months and the corresponding median PFS was 26.5 months (Fig. 2). Multivariable Cox regression analysis (Table 2) found that elderly patients had a 37% higher risk of disease progression or death (HR = 1.37; 95% CI:1.13-1.66) compared with patients < 65 years of age, and patients with high-risk cytogenetics had a 44% higher risk of disease progression or death (HR = 1.44; 95% CI:1.19-1.75) compared with standard-risk patients. ISS disease stage II and III patients had 31% and 37% higher risks of disease progression or death (HR = 1.31; 95% CI:1.06-1.63 and HR = 1.37; 95% CI:1.10-1.70), respectively, compared with ISS disease stage I patients, and patients with a worse ECOG PS score (≥2) also had a 49% higher risk of disease progression or death (HR = 1.49; 95% CI:1.22-1.81) compared with functionally active patients.

**Table 2:** Multivariable Cox regression analysis of associations between demographic and clinical characteristics and PFS

Characteristic	HR	95% CI lower limit	95% CI upper limit
Age at index, years			
< 65	1	–	–
65 to < 70	0.946	0.750	1.192
70 to < 75	1.079	0.870	1.338
≥ 75	1.370	1.133	1.658
Gender			
Female	0.955	0.825	1.104
Male	1	–	–
Race			
Black or African American	1.089	0.895	1.326
Asian or other race	1.069	0.862	1.325
White	1	–	–
Cytogenetic risk			
High	1.441	1.185	1.751
Standard	1	–	–
RI^a^	1.156	0.920	1.453
Diabetes	1.000	0.768	1.301
ISS disease stage			
I	1	–	–
II	1.310	1.055	1.626
III	1.370	1.103	1.702
ECOG PS score			
0	1	–	–
1	1.033	0.862	1.237
≥ 2	1.490	1.224	1.814
Year of index date	1.027	0.966	1.092
Time from initial diagnosis to index date, months	1.036	1.015	1.057

*PFS* progression-free survival*, HR* hazard ratio, *CI* confidence interval, *RI* renal impairment, *ICD-9-CM* International Classification of Disease, 9th Revision, Clinical Modification; *ICD-10-CM* International Classification of Disease, 10th Revision, Clinical Modification; *ISS* International Staging System, *ECOG PS* Eastern Cooperative Oncology Group performance status

^a^Acute RI, defined as patients with a serum creatinine > 2 mg/dL on the closest date to the index date or having diagnosis codes of ICD-9-CM 584.5-584.9, ICD-9-CM 586, ICD-10-CM N17.0-2, N17.8-9, and N19, measured ≤90 days prior to and ≤ 7 days after the index date

## Discussion

Our analysis among patients who received VRd as first LOT in the real-world setting demonstrated that the median PFS (26.5 months) was notably shorter than that observed in the SWOG S0777 clinical study of VRd versus Rd for the treatment of NDMM (median unstratified PFS for VRd, 43 months) [8]. Additionally, while ISS disease stage, ECOG PS score, and the proportion of patients with standard-risk cytogenesis were similar between studies, we found substantial differences in age and frailty of patients treated with VRd as first LOT between our real-world study and the SWOG S0777 clinical trial. In this current analysis, the majority of nontransplanted NDMM patients treated with VRd as first LOT were older and a greater proportion were frail compared to the population in the SWOG S0777 study (≥65 years of age: 64% in our study vs 38% in SWOG S0777; frail: 48% vs 21%) [8, 11]. Results from our present analysis and the SWOG S0777 clinical trial shed light on the differences between real-world use of treatment regimens and the patient populations enrolled in relevant clinical trials, which are often the basis for treatment guidelines. Specifically, based on the SWOG S0777 findings, the NCCN Guidelines include VRd as one of the preferred regimens in transplant-ineligible patients with NDMM in the United States [7]. Of note, patients in the real-world setting may have received VRd with a modified dosing schedule, differing from that in the SWOG S0777 study, for bortezomib and reduced dosing of lenalidomide and dexamethasone. This modified dosing schedule can be an alternative for patients with lower tolerance due to age, frailty, or other risk factors and to mitigate side effects often observed with bortezomib, such as peripheral neuropathy and thrombocytopenia. Physician familiarity with modifying the dose of the VRd regimen for an older or frail population seems to have expanded the demographics of patients who are considered eligible for up-front VRd [18–20]. In our study, however, we were unable to capture data on dosing and thus unable to confirm the association between demographics and dose reduction in these patients.

Contrary to observations from phase 1 and phase 2 clinical studies showing that VRd performed equally well in patients with high-risk cytogenetics and in patients with standard-risk cytogenetics [21], our study showed that high-risk cytogenetics were associated with a 44% higher risk of disease progression or death compared with standard cytogenetic risk (HR = 1.44; 95% CI: 1.19-1.75). Our findings are consistent with those from the SWOG S0777 study in which patients who received VRd and had high cytogenetic risk had median PFS of 38 months, which is numerically shorter than that of the overall population [8]. Additionally, our findings establish that elderly patients (specifically ≥75 years of age) still face a higher risk of disease progression or death than younger patients (HR = 1.37; 95% CI: 1.13-1.66), even with this preferred first-line regimen. This finding is consistent with the observation in the SWOG S0777 trial that median PFS in the VRd arm was shorter for the subgroup of patients > 75 years of age (39 months) than for the overall study population (43 months) [8]. The present analysis also shows that patients with higher ISS disease stage and worse ECOG PS score (≥2) are at an increased risk for disease progression or death. Regarding the observation that patients in this study had a rather short median duration of therapy of 5.5 months, it may be worth noting that durations of therapy in the real world may be shorter than those observed or specified in clinical trials, possibly, for instance, because of efforts to decrease treatment burden and reduce treatment-emergent neuropathy [22].

Our study offers real-world insights beyond the existing literature on transplant-ineligible patients with NDMM, including a European real-world study that evaluated patients who received bortezomib-based regimens as first LOT between June 1, 2015, and November 30, 2016 (VRd was approved for transplant-ineligible patients by the European Medicines Agency in 2019) [23, 24]. In that European analysis, the most common first-line bortezomib-based regimens were bortezomib in combination with melphalan and prednisone (*n* = 83; 35%), Vd (*n* = 82; 35%), and bortezomib in combination with cyclophosphamide and dexamethasone (*n* = 32; 13%), which are different from common practices in the United States. Real-world use of the VRd regimen was not included in the European study [23, 24], and, to our knowledge, VRd in transplant-ineligible NDMM has not been reported on by any other real-world study. We also note that, in addition to VRd, the other preferred regimen recommended by the NCCN Guidelines for primary therapy for nontransplant patients is D-Rd [7]. Pivotal clinical studies, including the phase 3 MAIA study, showed that D-Rd provides significant clinical benefit in transplant-ineligible patients with NDMM [10]. Additionally, the PFS benefit of D-Rd versus Rd was also observed in a subgroup analysis of MAIA among frail patients, who constituted 46.3% of the overall population and had baseline median age of 77.0 years [25].

Findings of the present study should be interpreted from the perspective of certain limitations. First, the Flatiron data used in this study are generated from real-world clinical practice, which may be subject to miscoding and errors. Data on ECOG PS score, ISS disease stage, and medication dosing are missing for some patients. Second, Flatiron is derived from an oncology EHR, and data from medical records outside of patients’ oncology care may be incomplete. In addition, some ICD codes might not have been finalized. As a result, baseline variables such as frailty score and diabetes diagnosis are likely to be underestimated, and, hence, the results based on these variables need to be interpreted with caution. Likewise, information about patient treatment outside of the specific cancer care site may not be captured in structured EHR data and, in turn, may impact the accuracy of the general comorbidity measures reported. Lastly, data are primarily from US community oncology practices, and results may not be generalizable to other populations.

## Conclusions

In this real-world analysis of nontransplanted patients who received VRd as first LOT for NDMM, the median PFS was 26.5 months, which was markedly shorter than that observed in the pivotal phase 3 SWOG S0777 study (43 months). Patients in our real-world analysis were older and a higher proportion were frail, compared with the SWOG S0777 study. The proportions of patients with ISS disease stage I or II, good functional status with an ECOG PS score of 0 or 1, and standard-risk cytogenetics were similar between the two studies. The efficacy of the VRd regimen in the real-world setting was not uniform across subgroups; a significantly higher risk of disease progression or death was associated with older age, high-risk cytogenetics, worse disease stage, and worse ECOG PS score. Substantial differences between the clinical study setting and real-world effectiveness could be attributed to differences observed in characteristics of patients being prescribed VRd in actual clinical practice. Continued exploration and discussion of differences between clinical trial participants and real-world use of this regimen are warranted and may inform when alternative treatment regimens should be explored for some patients.

## Supplementary Information

**Additional file 1: Supplementary Table 1.** Frailty assessment based on the FIRST study.

## Data Availability

The data sharing policy of Janssen Pharmaceutical Companies of Johnson & Johnson is available at https://www.janssen.com/clinical-trials/transparency. As noted on this site, requests for access to the study data can be submitted through Yale Open Data Access (YODA) Project site at https://yoda.yale.edu.

## Abbreviations

CI: Confidence interval
CRAB: Hypercalcemia, renal impairment, anemia, and bone disease
D-Rd: Daratumumab in combination with lenalidomide and dexamethasone
ECOG PS: Eastern Cooperative Oncology Group performance status
EHR: Electronic health record
HR: Hazard ratio
HSCT: Hematopoietic stem cell transplant
ICD-9-CM: International Classification of Disease, 9th Revision, Clinical Modification
ICD-10-CM: International Classification of Disease, 10th Revision, Clinical Modification
ISS: International Staging System
LOT: Line of therapy
MM: Multiple myeloma
NDMM: Newly diagnosed multiple myeloma
PFS: Progression-free survival
Rd.: Lenalidomide in combination with dexamethasone
RI: Renal impairment
SD: Standard deviation
SWOG: Southwest Oncology Group
Vd: Bortezomib in combination with dexamethasone
VRd: Bortezomib in combination with lenalidomide and dexamethasone
