# Supplementary material for: Real-world patient characteristics and treatment outcomes among nontransplanted multiple myeloma patients who received Bortezomib in combination with Lenalidomide and Dexamethasone as first line of therapy in the United States
Source: BMC Cancer. 2022 Aug 18;22:901. doi: 10.1186/s12885-022-09980-9 (PMC9386920; doi:10.1186/s12885-022-09980-9)
Supplement: Supplementary file 1 — Additional file 1: Supplementary Table 1. Frailty assessment based on the FIRST study. [file 12885_2022_9980_MOESM1_ESM.pdf]

Online Supplementary Material for Medhekar et al. Real-world Patient Characteristics and Treatment Outcomes Among Nontransplanted Multiple Myeloma Patients Who Received Bortezomib in Combination With Lenalidomide and Dexamethasone as First Line of Therapy in the United States

This file provides supplementary methods.

Supplementary Table 1 Frailty assessment based on the FIRST study

Category	Score
Age	
≤75 years	0
76-80 years	1
>80 years	2
Charlson Comorbidity Index[a]	
≤1	0
>1	1
ECOG PS score	
0	0
1	1
≥2	2
Sum of scores	
Non-frail	0-1
Frail	≥2

ECOG PS Eastern Cooperative Oncology Group performance status

[a]The Charlson Comorbidity Index was calculated based on the definition provided by Palumbo et al with slight modifications [1]. One point each was assigned for myocardial infarction (history of definite or probable myocardial infarction, including electrocardiography changes and/or enzyme changes), congestive heart failure, peripheral disease (includes aortic aneurysm ≥6 cm), cerebrovascular disease (cerebrovascular accident with mild or no residua or transient ischemic attack), dementia, chronic obstructive pulmonary disease, connective tissue disease, peptic ulcer disease, mild liver disease (chronic hepatitis or cirrhosis without portal hypertension), and uncomplicated treated diabetes mellitus without end-organ damage (excludes diet-controlled alone); 2 points each were assigned for hemiplegia, moderate or severe renal disease (excludes renal disease due to multiple myeloma that resolved after the start of study treatment), diabetes with end-organ damage (retinopathy, neuropathy, nephropathy, or brittle diabetes), tumor without metastasis (exclude if >5 years from diagnosis and excludes benign tumor, basal cell carcinoma, squamous cell carcinoma of the skin, or carcinoma in situ), leukemia (acute or chronic), and lymphoma; 3 points were assigned for moderate or severe liver disease; and 6 points each were assigned for metastatic solid tumor and acquired immunodeficiency syndrome (not just human immunodeficiency virus positive).

ECOG PS Eastern Cooperative Oncology Group performance status.

Adapted from Facon et al [2], which is licensed under the Creative Commons Attribution 4.0 International License (http://creativecommons.org/licenses/by/4.0/).

References

1. Palumbo A, Bringhen S, Mateos MV, et al. Geriatric assessment predicts survival and toxicities in elderly myeloma patients: an International Myeloma Working Group report. Blood. 2015;125:2068-74.

2. Facon T, Dimopoulos MA, Meuleman N, et al. A simplified frailty scale predicts outcomes in transplant-ineligible patients with newly diagnosed multiple myeloma treated in the FIRST (MM-020) trial. Leukemia. 2020;34:224-33.
